# Insect Pollinators in Iowa Cornfields: Community Identification and Trapping Method Analysis

**DOI:** 10.1371/journal.pone.0143479

**Published:** 2016-07-26

**Authors:** M. J. Wheelock, M. E. O’Neal

**Affiliations:** Department of Entomology, Iowa State University, Ames, IA, 50011, United States of America; Central China Normal University, CHINA

## Abstract

Availability of mass flowering plants in landscapes dominated by agriculture can have a strong positive impact on the density of generalist, native pollinators. Row-crop production in Iowa accounts for 75% of the arable acres, with corn, *Zea mays*, representing the majority of hectares planted. To date, there has been no description of the insect pollinator community found within Iowa cornfields. We report a field study to determine the optimal sampling methodology to characterize the community of insect pollinators within cornfields. During 2012 and 2013, 3,616 insect pollinators representing 51 species were captured using bee bowls, and 945 individuals representing 10 species were captured using sticky cards. We examined the effects of trap type, height, and bowl color on the described community. Bee bowls captured a more abundant and species rich community than sticky cards with all species captured on sticky cards also present in bee bowls. Traps deployed at the height of the tassels describe a more abundant and species rich community of pollinators than traps at ear height (2x as many individuals) or ground height (4x as many individuals). Blue bowls captured more bees than white (2.75x as many individuals) or yellow bowls (3.5x as many individuals); and yellow bowls captured more flies than white (2x as many individuals) or blue (2.3x as many individuals). To provide the most complete description of the community of insect pollinators using cornfields as a resource, we suggest sampling-using bee bowls at the height of the tassels using all three bee bowl colors.

## Introduction

Fragmented landscapes devoted to annual crop production generally have lower insect biodiversity, including pollinators, than natural landscapes do [1]. However, mass flowering crops can have a strong impact on the density of generalist pollinators, such as bumblebees (*Bombus* spp.) [2]. Iowa’s landscape is dominated by agriculture, with 75% of arable acres planted in row crop production, primarily corn (*Zea mays)* and soybean (*Glycine max)*[3]. Both corn and soybean may be considered mass flowering crop species. While neither requires insects for pollination[4], both may provide resources for pollinators that forage within them.

Recently Gill described a robust community of at least 50 species of insect pollinators visiting Iowa soybean fields [5]. This community was composed of mostly of solitary, ground nesting bees. Social bees, such as bumblebees and the honey bee (*Apis mellifera)* were rarely detected, represented only 0.005% of individuals collected. The most abundant species described by Gill included *Agapostemon virescens*, *Lasioglossum* (*Dialictus)* spp., *Melissodes bimaculata* and *Toxomerous marginatus* [5]. The most commonly collected species were also found carrying soybean pollen. This suggests that soybean flowers are a source of nectar and/or pollen for several native social and solitary bees. Additionally anthophilious flies exploit these crops for resources, especially syrphid adults that consume nectar from a variety of flowering species [6].

Corn is wind pollinated and does not require insect mediated pollination to set seed; however, like soybean, it may be a resource for pollinators. Recently Gardiner et al examined the implication of three different bio-fuel crops, corn, switch grass, and mixed prairie on beneficial arthropods in an agricultural landscape [7]. To assess the pollinator community, bee bowls were placed on the ground of cornfields for intervals of 48 h during a growing season. They reported 213 individuals representing 42 species. Gardiner is the first study to examine the potential community of bee pollinators that may use cornfields in Michigan as a resource. It is possible that their method under-represented the abundance and diversity of pollinators within cornfields as the height and position of the bee bowls have an effect on capture [8]. Since bee bowls were left on the ground they may not have captured insects visiting the tassels or the silks.

To date, there has been no description of insect pollinator communities that visit Iowa cornfields. Such baseline data can inform conservation and management decisions, and be used to assess what species may be at risk from agronomic practices. Describing the community of insect pollinators using cornfields as a resource requires the establishment of a sampling method that will allow for comparisons with future studies. Passive methods of sampling, such as sticky traps and bee bowls have been used to describe pollinators in agro-ecosystems [9]; however, sampling methods often need to be modified for optimal use within a specific crop, especially if a unique guild of insects is targeted [10]. We conducted a field study to determine the optimal sampling method to characterize the community of pollinators within cornfields.

## Materials and Methods

### Sampling sites

During 2012 and 2013, insects were sampled at three Iowa State University research farms. All farms were located in central Iowa, at least 2 km from each other (Table 1). We began sampling one week prior to the occurrence of tassels (VT) and continued to milk stage (R3)[11]. In 2012 sampling occurred from 3-July-9-August-2012 and from 16-July-23-August-2013. Growth stage was estimated at each sampling date, taking special note when tassels emerged and when pollen shed was complete. Pollen shed was estimated by shaking tassels of five random plants and visibly looking for pollen. No specific permissions were required to sample insect pollinators at these university research farms as no endangered/ protected species were involved in this study.

**Table 1:** Iowa cornfields surveyed for pollinators.

Year	County	Coordinates
2012	Boone	42°00'05.69''N 93°47'19.72''W
	Story	42°00'08.54''N 93°39'32.57''W
	Story	41°58'54.94''N 93°38'38.41''W
2013	Boone	42°00'05.69''N 93°47'19.72''W
	Story	42°06'23.65''N 93°35'23.79''W
	Story	42°00'08.54''N 93°39'32.57''W

### Collection methods

Unbaited Pherocon^®^ AM yellow sticky cards (YSC, Trécé Incorporated) and bee bowls (BB) adapted from Droege were used to sample pollinators [9]. Bee bowls (3.25oz. SOLO^®^ brand white plastic soufflé cups, Food Service Direct, Hampton, VA, USA) were painted fluorescent yellow and fluorescent blue (East Coast Guerrra Paint and Pigment, New York, NY, USA) or left white and filled halfway with a soapy water solution. We choose YSC as they are recommended for sampling insect pests in cornfields and are commercially available [12]. Bee bowls were selected because they are considered effective for sampling pollinators in a variety of ecosystems. In addition, BB have been used in corn [7] and soybean [5] to describe pollinator communities.

**Fig 1:**
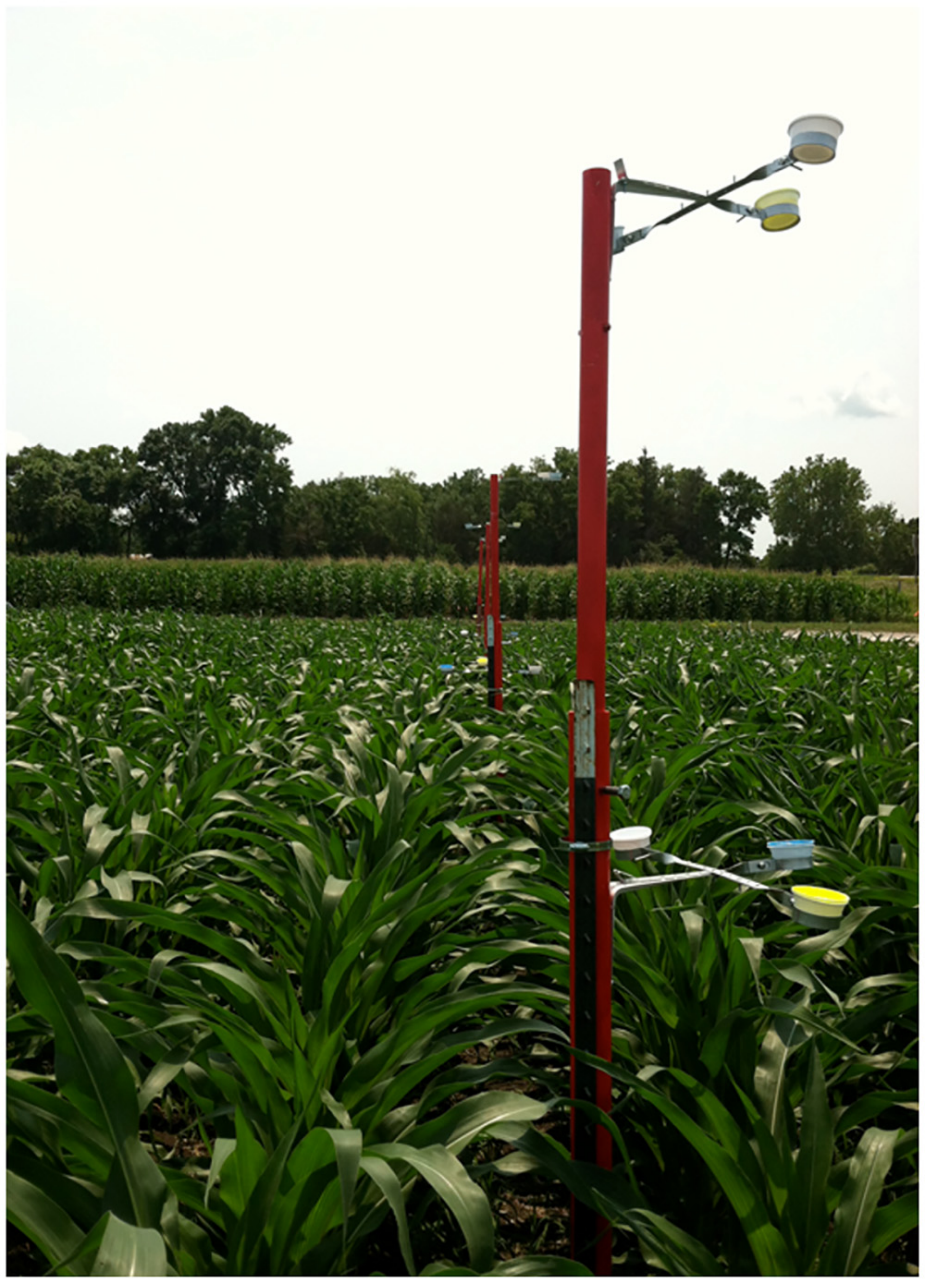
Bee bowl stand used to sample insect pollinators in cornfields, raised to maximum height of 2.74 m. Image was taken in early June prior to sampling to illustrate how traps function; traps were obscured by the corn plants once sampling began.

**Fig 2:**
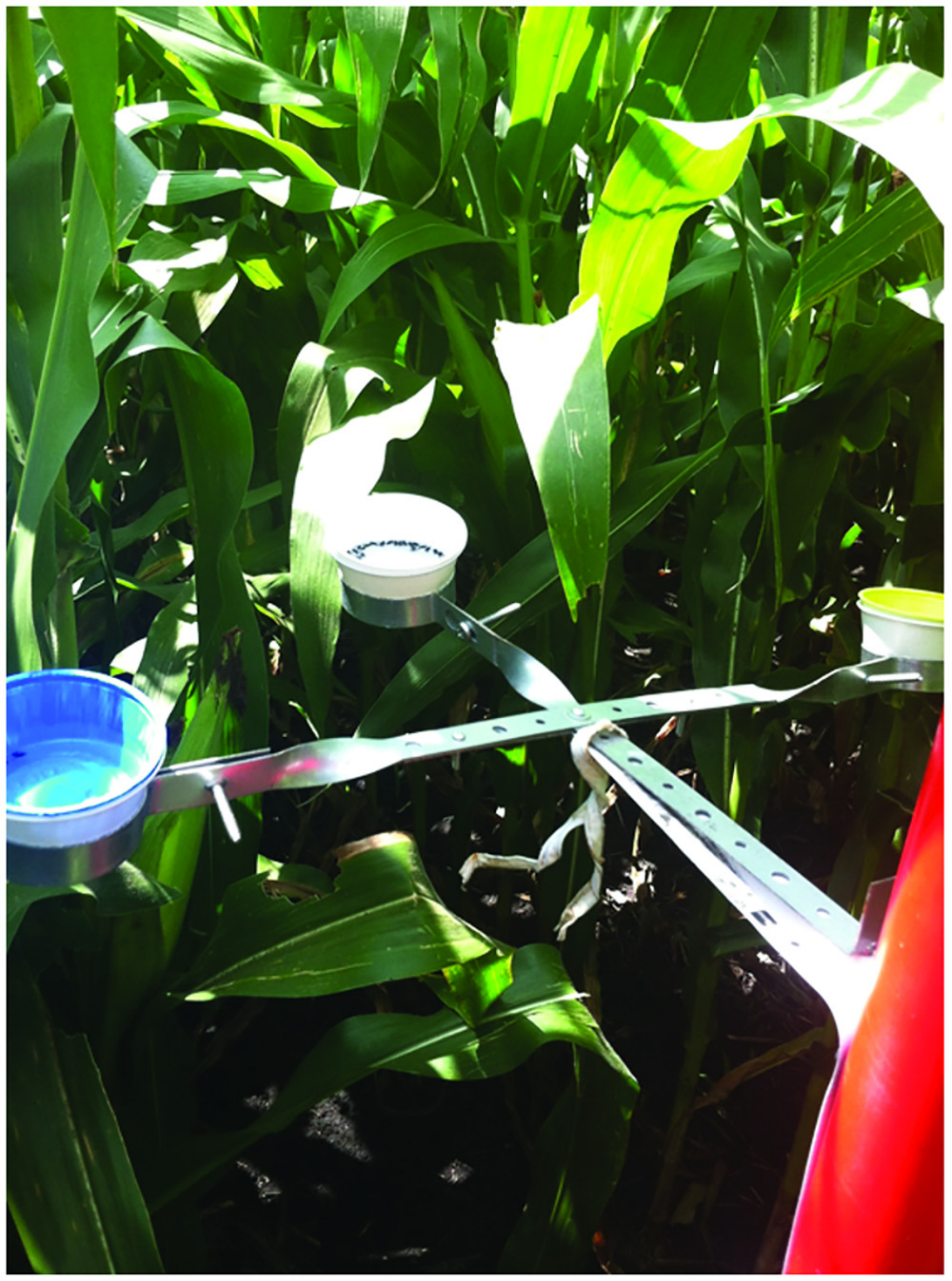
A ‘bowl-unit’ at ear height contained one bowl of each color (yellow, white, and blue) made from three hangers attached to a wall shelf bracket.

Telescoping poles were constructed so that BB and YSC could be set at multiple heights next to a mature corn plant (Fig 1). Each trap consisted of two 1.52 m sections of schedule 40 PVC. One section was 3.8 cm in diameter, the other 5.1 cm in diameter, allowing the smaller section to fit within the larger one. When combined, these two sections reached a maximum height of 2.74 m. Bee bowls were attached to the pole by connecting three galvanized steel pipe-hangers to a shelf bracket (Fig 2), so that one of each color-white, yellow, and blue-were present at each height. We refer to a set of three BB (one of each color) as a ‘bowl unit’ hereafter. Bowl units were attached at ground height, ear height, and tassel height. The bowl unit at ground height and ear height were fixed at 0.308 m and 1.22 m, respectively. The bowl unit at tassel height was never enclosed by the canopy and was adjusted as the corn plant grew from a starting height of 1.5 m to a maximum of 2.74 m. The same pole was used to deploy YSC, with a single YSC attached at each of the three heights.

**Fig 3:**
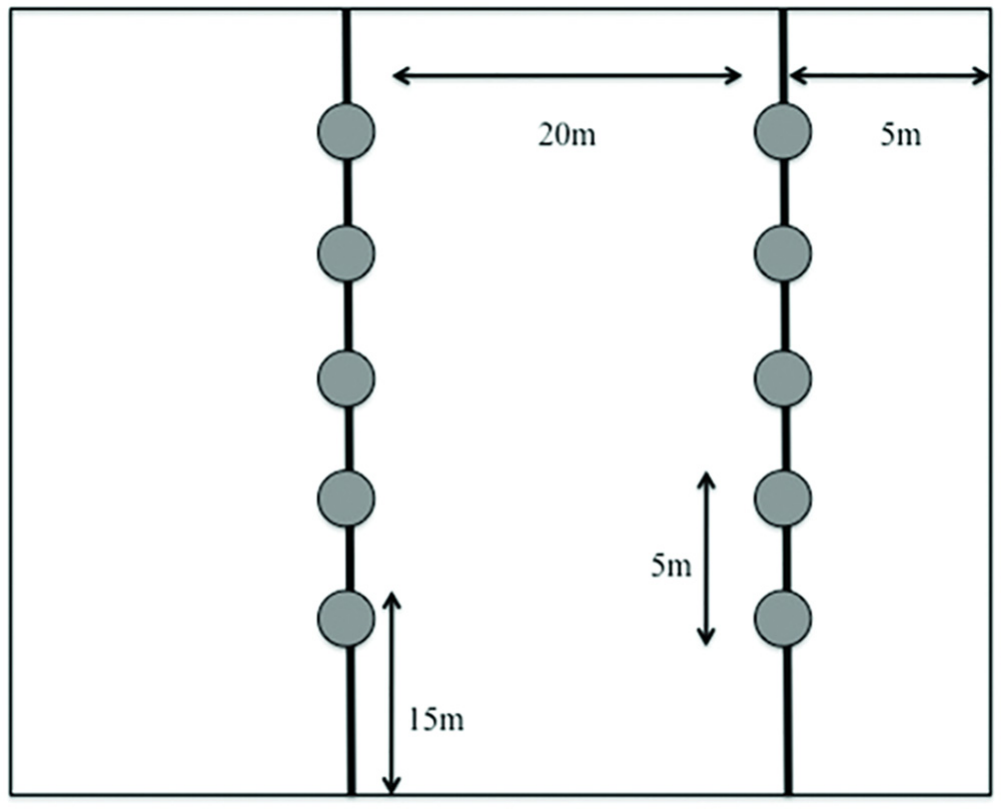
Diagram depicting the location within a cornfield where bees were sampled; circles represent location of traps.

Two parallel transects were established at each farm; one located 5 m into the field from the nearest edge and a second located 20 m farther in field from the first transect. Each transect started 15 m into the field and consisted of 5 traps spaced 5m apart (Fig 3). At each farm, 90 BB (3/height/trap) were deployed for 24 h once a week during this period. After BB were collected, 30 YSC per farm (1/height/trap) were deployed and left in the field for 5 d.

### Specimen processing and identification

Insects captured using BB were processed according to the methods outlined by Droege [9], and specimens identified using the Discover Life key [13]. Specimens were identified to species with the exception of bees in the genus *Lasioglossum*, which were grouped by sub-genera. Pollinating flies (Syrphidae, Tachinidae, Dolichopodidae, or Bombyliidae) were grouped by morpho-species within each family. Specimens from YSC were identified to the lowest taxonomic unit possible based on condition.

### Pollen analysis

To determine if bees captured in BB were foraging in the field, we examined captured bees for corn pollen. We examined a subset of bees that had visible pollen loads on the body during the period when corn pollen was being shed in a given field. Pollen grains were combed from the scopa, rinsed in ethanol, and then slide mounted using glycerin jelly for identification with a light microscope. Pollen grains removed from bees were compared to light micrographs obtained from the USDA pollen library [14] and to reference slides containing corn pollen obtained from plants at each study farm.

### Data analysis

Abundance of species and morpho-species were recorded for each sample. To meet the assumptions of multivariate normality a log transformation of abundance data was performed. Abundance data were characterized by a high number of zero values; therefore 0.5 was added to each cell such that data could be log transformed. A three-way multivariate analysis of variance (MANOVA) was conducted to test the hypothesis that the abundance of bees and flies captured in bowls varied by the following factors: bowl color, trap height, and farm. The statistical model also contained the interactions of height*bowl color and height*farm as multivariate fixed effects. Significant effects in the MANOVA were examined using separate analysis of variance (ANOVA) tests to understand the effect size separately for both bees and flies using the post-hoc Tukey Honest Significant Differences (Tukey HSD) test.

To determine if we had fully captured the pollinators visiting cornfields, we constructed species accumulation curves using the vegan package in R [15] with data from the bee bowls. The species included all insects considered pollinators or at least. We reported these curves by trap height and bowl color.

## Results

During the course of this study a total of 3,616 insects were captured using BB and 945 were captured using YSC (Table 2). When these data were combined, the pollinator community found in Iowa cornfields contained at least 51 taxonomic units (species and morpho-species). Bees accounted for 60% and flies accounted for 40% of total abundance summed across trapping types. There were at least 36 species of bee, 9 species of syrphid fly, and 6 morpho-species from other fly families. The bee community was composed of mostly solitary, ground nesting, bees.

**Table 2:** Abundance of pollinator by taxa and trapping method for 2012 (2013).

Taxa		Sampling Method	
**HYMENOPTERA**		**Bee Bowls**	**Sticky Cards**
**Andrenidae**			
	*Andrena wilkella* (Kirby)	2 (2)	0 (0)
	*Calliopsis andreniformis* (Smith)	1 (7)	0 (0)
**Apidae**			
	*Anthophora bomboidies*	0 (1)	0 (0)
	*Apis mellifera* L.	5 (12)	2 (2)
	*Bombus auricomus*	0 (1)	0 (0)
	*Bombus bimaculatus* (Cresson)	0 (1)	0 (0)
	*Bombus fraternus*	0 (1)	0 (0)
	*Bombus griseocollis*	0 (1)	0 (0)
	*Bombus impatiens*	2 (2)	0 (0)
	*Ceratina* Spp.	0 (0)	1 (0)
	*Melissodes agilis*	14 (25)	0 (0)
	*Melissodes bimaculata* (Lepeletier)	272 (474)	0 (0)
	*Melissodes communis*	22 (14)	0 (0)
	*Melissodes druriella* (Kirby)	0 (10)	0 (0)
	*Melissodes nivea*	0 (1)	0 (0)
	*Melissodes trinodus* (Robertson)	13 (35)	0 (0)
	*Nomada* Spp.	0 (0)	0 (0)
	*Svastra atripes*	0 (1)	0 (0)
	*Svastra obliqua*	0 (6)	0 (0)
**Chrysididae**			
	Chrysididae spp.	0 (1)	0 (0)
**Colletidae**			
	*Hylaeus affinis* (Smith)	2 (4)	2 (2)
**Halictidae**			
	*Agapostemon texanus*	28 (68)	0 (0)
	*Agapostemon virescens* (F.)	124 (290)	0 (0)
	*Augochlora pura* (Say)	3 (29)	0 (0)
	*Augochlorella aurata* (Smith)	46 (32)	0 (0)
	*Augochloropsis metallica* (F.)	0 (7)	0 (0)
	*Dieunomia heteropoda*	2 (6)	0 (0)
	*Dieunomia triangulifera*	1 (0)	0 (0)
	*Halictus confusus* (Smith)	22 (33)	0 (0)
	*Halictus ligatus* (Say)	6 (28)	0 (0)
	*Halictus parallelus*	4 (0)	0 (0)
	*Halictus rebicondus* (Christ)	6 (11)	0 (0)
	*Halictus tripartitus* (Cockerell)	1 (2)	0 (0)
	*Lasioglossum (Dialictus)* spp.	268 (762)	17 (4)
	*Nomia universitatis*	0 (2)	0 (0)
	*Xenoglossa strenua*	0 (2)	0 (0)
**Megachilidae**			
	*Megachile relativa*	0 (2)	0 (0)
	*Megachilie rotundata* (F.)	0 (1)	0 (0)
**DIPTERA**			
**Bombyliidae**		0 (1)	0 (0)
**Calliphoridae**		11 (22)	5 (1)
**Dolichopodidae**		77 (53)	169 (43)
**Tachinidae**			
	Tachinidae morphospecies 1	19 (2)	0 (0)
	Tachinidae morphospecies 2	5 (55)	0 (0)
	Tachinidae morphospecies 3	1 (44)	0 (0)
	Tachinidae Undet.	0 (0)	17 (225)
**Syrphidae**			
	*Eristalis transversa*	0 (3)	0 (0)
	*Eristalis* spp. 1	2 (5)	0 (0)
	*Helophilu*s spp.	0 (2)	0 (0)
	*Melanostoma mellinum* (L.)	0 (1)	0 (0)
	*Platycheirus* spp.	0 (2)	0 (0)
	*Sphaerophoria* spp.	1 (18)	0 (0)
	Syrphus spp 1	2 (4)	0 (0)
	*Toxomerous geminatus* Say	5 (18)	19 (0)
	*Toxomerous marginatus* Say	32 (514)	16 (0)
	Syrphidae Undet.	0 (0)	1 (419)
TOTALS			
**Hymenoptera**		**845 (1872)**	**22 (8)**
**Diptera**		**155 (744)**	**227 (688)**
**GRAND TOTAL**		**1000 (2616)**	**249 (696)**

Yellow sticky cards captured 30 bees representing 5 species across all dates, heights, and farms. We observed more flies on YSC than in bee bowls, capturing 915 individuals across all dates, heights, and farms. No significant differences in bee species richness or abundance were detected among the YCS placed at different heights. Bee bowls were a more effective tool at describing the community of insect pollinators in Iowa cornfields (Table 2), as more individuals and more species of insect pollinators were collected using BB (2,717 individual bees representing ~36 taxonomic units and 899 individual flies representing ~15 taxonomic units) compared to YSC. Therefore, the following analysis will focus on data obtained from BB samples.

Bee bowls captured a total of 2,717 individual bees (representing ~36 taxonomic units) and 899 individual flies (representing ~15 taxonomic units) across all farms and dates. In total 3,240 bee bowls were deployed of which 1,105 bowls contained at least one individual, resulting in an overall success rate of 34%. Bees dominated the bee bowl catch, accounting for 75% of individuals captured. The most abundant species did not differ among years. The most abundant bee species captured were *Lasioglossum (Dialictus)* (28%), *Melissodes bimaculata* (20%), and *Agpostemon virescens* (11%). The most abundant flies were *Toxomerus marginatus* (15%) and flies belonging to Dolichopodidae (4%).

### Summary of bee bowl sampling method

We observed significant main effects for bowl color, height, and farm within the three-way MANOVA as well as significant effects for the interactions of height*bowl*color and height*farm (Table 3). Analysis of variance fit statistics were examined for the significant multivariate main effects on log bee abundance and log fly abundance separately. Bee abundance was significantly affected by height, color, farm, and the interaction of height*color. The interaction of height*farm was not significant. For fly abundance only the main effects were significant; the interactions of height*color and height*farm were not significant (Table 4).

**Table 3:** MANOVA fit statistics height, color, and farm on bee and fly abundances in bee bowls deployed in Iowa cornfields.

Effect	Df	Wilks λ	ApproxF	NumDf	DenDf	Pr>F
Height^a^	2	0.552	53.203	4	616	0.0001
Color^b^	2	0.571	49.680	4	616	0.0001
Farm^c^	2	0.740	24.967	4	616	0.0001
Height*Color	4	0.942	2.297	8	616	0.019
Height*Farm	4	0.944	2.254	8	616	0.022
Residuals	309					

^a^ Traps were set at ground (0.308m), ear (1.22m), and a variable tassel height (1.5m-2.74m).

^b^ One bowl colored yellow, white, and blue were present at each trapping height.

^c^ Three farms were sampled in each year. Farms were all located in central Iowa with each at least 2km from one another.

**Table 4:** ANOVA fit statistics for the effects of height, color, and farm separately for bee and fly abundance from bee bowl deployed in Iowan cornfields.

*Log Bee Abundance*				
Effect	Df	Mean Sq.	F-Value	Pr>F
Height^a^	2	17.415	105.0606	0.0001
Color^b^	2	9.4544	57.0366	0.0001
Farm^c^	2	8.0816	48.7544	0.0001
Height*Color	4	0.6012	3.6268	0.0067
Height*Farm	4	0.3895	2.3496	0.0543
Residuals	309	0.1658		
*Log Fly Abundance*				
Effect	Df	Mean Sq.	F-Value	Pr>F
Height^a^	2	1.765	9.6192	0.0001
Color^b^	2	3.799	20.7038	0.0001
Farm^c^	2	4.072	22.1898	0.0001
Height*Color	4	0.067	0.3667	0.8323
Height*Farm	4	0.310	1.6869	0.1528
Residuals	309	0.1835		

^a^ Traps were set at three different heights: ground (0.308m), ear (1.22m), and a variable tassel height (1.5m-2.74m).

^b^ One bowl of each color, yellow, white, and blue, were present at each trapping height.

^c^ Three farms were sampled in each year. Farms were all located in central Iowa with each at least 2km from one another.

Bowl units at tassel height captured significantly more bees on average than bowl units at ear (2x as many) and ground height (4x as many; Table 5). Significant differences in bee abundace were detected, with blue bowls capturing more than yellow (3.5x as many) or white (2.75x as many). There was no significant difference between yellow or white bowls. There were significant differences in fly abundace for levels of height and bowl color as well. Significant differences among levels of height were detected, with bowl units at ear height capturing more than bowl units at ground height (1.75x as many) and capturing more than bowl units at tassel height (1.5x as many). There were no detectable differences between bowl units at tassel height and bowl units at ground height. Flies were not equally attracted to each bowl color. Fly abundance varied across the three levels of bowl color with yellow bowls capturing more on average than white (2x as many) or blue bowls (2.3x as many). There were no detectable differences between blue or white bowls.

**Table 5:** Tukey Honest significant differences for varying levels of height and color for bee and fly abundance from bee bowls deployed in Iowan cornfields.

*Log bee abundance by height*				
Contrast	Diff	Lwr	Upr	p adj
Tassel-Ground	0.79	0.66	0.92	0.0001
Ear-Ground	0.53	0.40	0.66	0.0001
Tassel-Ear	0.26	0.13	0.39	0.0001
*Log bee abundance by bowl color*				
Contrast	Diff	Lwr	Upr	p adj
Blue-White	0.56	0.43	0.69	0.0001
Blue-Yellow	0.44	0.31	0.57	0.0001
Yellow-White	0.12	0.00	0.25	0.0680
*Log fly abundance by height*				
Contrast	Diff	Lwr	Upr	p adj
Ear-Ground	0.25	0.12	0.39	0.0001
Ear-Tassel	0.16	0.20	0.29	0.0190
Tassel-Ground	0.10	-0.04	0.23	0.2280
*Log fly abundance by bowl color*				
Contrast	Diff	Lwr	Upr	p adj
Yellow-Blue	0.36	0.23	0.50	0.0001
Yellow-White	0.29	0.15	0.42	0.0001
White-Blue	0.08	-0.06	0.21	0.3874

**Fig 4:**
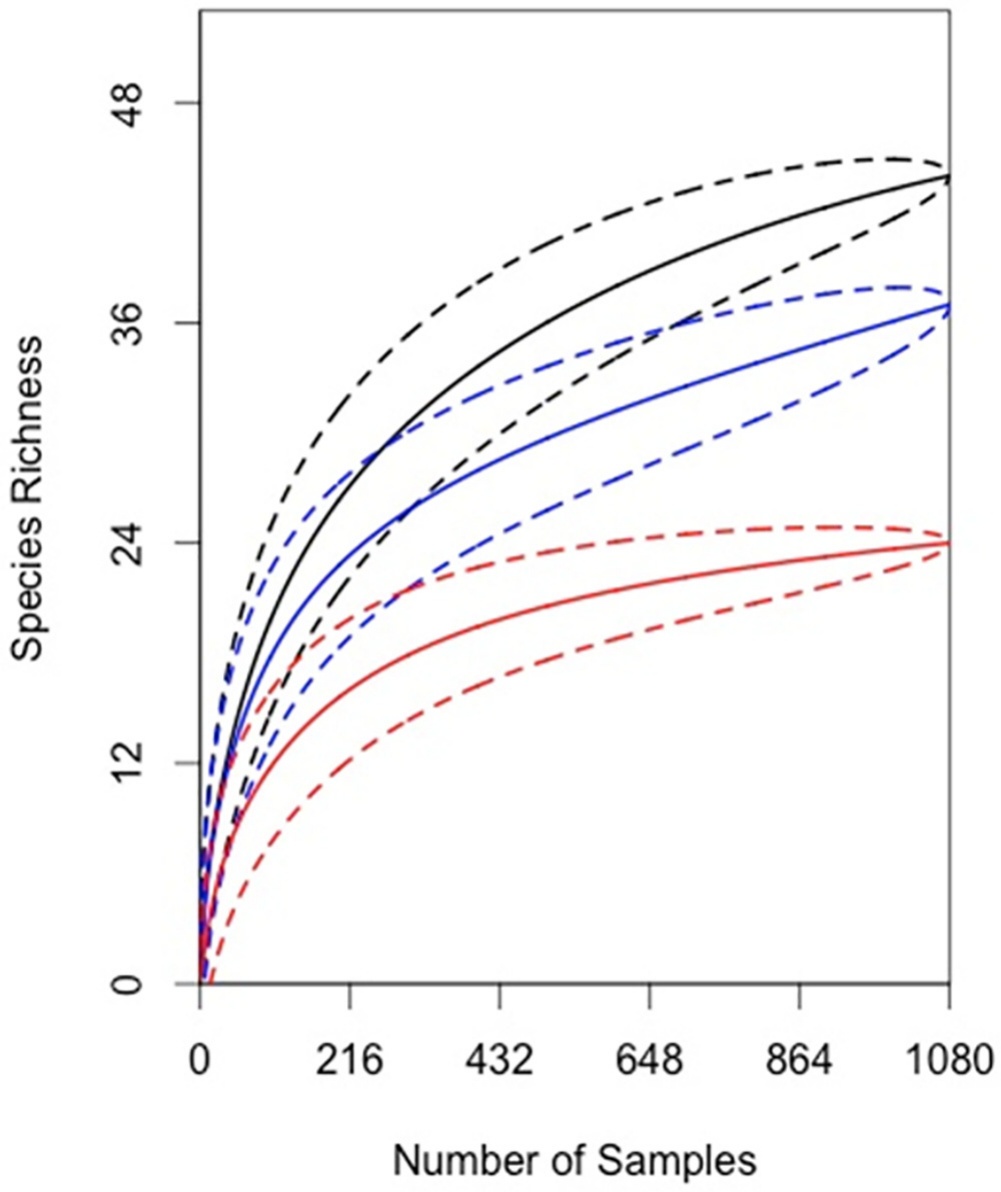
Species accumulation curves generated from samples collected from bee bowls in 2012 and 2013. Curves represent the accumulation of both bee and fly species. The black curve corresponds to traps at tassel height; the blue curve corresponds to traps at ear height; and the red curve corresponds to traps at ground height. The dashed lines about the curves represent the 95% confidence interval.

Not only did the sampling methodology affect the abundance of insect pollinators captured, it also affected the species richness of the captured community. Species richness was not equally distributed across all sample heights. Bowl units at tassel height captured a total of 44 species, bowl units at ear height captured 37 species, and bowl units at ground height captured 24 species. We observed limited overlap among the species accumulation curves developed from the three heights (Fig 4). Each curve approached an asymptote, suggesting that increasing the sample effort will not increase the likelihood of capturing novel taxa. In addition the curves suggest that the richness at each height are significantly different from one another, as the confidence intervals eventually did not overlap as each curve approached an asymptote. These curves suggest that traps placed at the height of the tassels collected the most species for the same amount of sampling effort.

While pollinators captured at tassel height represent 87% of all species captured in BB, a small but unique suite of species was captured at ear and ground heights. The following speices were captured only at ear height: *Bombus auricomus*, *B*. *fraternus*, a Chrysididae species, *Eristalis transversa*, and a *Platycheirus* species. *Calliopsis andreniformis* and *Melanostoma mellinum* were only captured at ground height. We captured a total of eight *C*. *andreniformis*, while the remaining species that were found only at ear or ground height were represented by single specimens.

**Fig 5:**
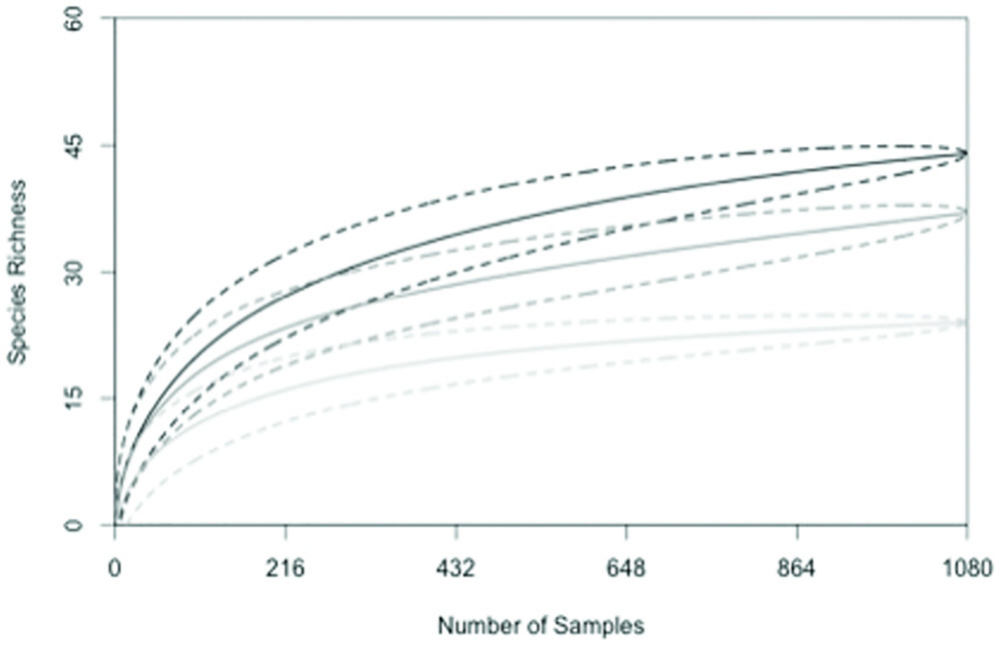
Species accumulation curves generated from samples collected from bee bowls in 2012 and 2013. Curves represent the accumulation of both bee and fly species. The black curve corresponds to samples collected from blue bowls; the medium grey curve corresponds to samples collected from yellow bowls and the light grey curve corresponds to samples collected from white bowls. The dashed lines about each curve represents the 95% confidence interval.

When species accumulation curves were examined by bowl color we observe a similar pattern. There is limited overlap among the curves generated by bowl color (Fig 5). Each is approaching an asymptote suggesting that increased sampling with bowls of that color is not likely to increase the likelihood of capturing novel taxa. The asymptote was lowest and most quickly reached for white bowls. The curves suggest that blue and yellow bowls collected the most species for the same amount of sampling effort.

Blue bowls captured 82% of all pollinator taxa in this experiment. Eight species were only found in blue bowls: *Dieunomia heteropoda*, *Nomada* sp., *Xenoglossa strenua*, *anthophora bomboidies*, *Nomia universitatis*, *Melissodes nivea*, and Bombyliidae. Yellow bowls captured 74% of all pollinator taxa with six novel taxa: *Bombus auricomus*, *B*. *griseocollis*, *B*. *fraternus*, *Melanostoma mellinum*, and *Eristalis transversa*. White bowls captured only 60% of the pollinator taxa with only one novel species, *Bombus impatiens*.

### Pollen Analysis

Of 1,782 female bees collected during pollen shed, 162 were carrying visible amounts of pollen. Of the bees with visible amounts of pollen, we identified corn pollen alone or intermixed with pollen grains from other plant species on 80 bees. These 80 bees represented five species. The most frequently captured *Lasioglossum (Dialictus)*, *Melissodes bimaculata*, and *Agpostemon virescens* were identified as carrying corn pollen. In addition we identified two more Apidae species carrying corn pollen: *Apis mellifera* and *Melissodes communis*.

## Discussion

We observed that sampling methodology affects the community of pollinators described in Iowa cornfields. Trap type had a significant effect on the community of insect pollinators collected with BB collecting a more abundant and diverse community of pollinators than YSC. Trap height also significantly affected the described community. Traps deployed at the height of the tassels describe a more abundant and species rich community of pollinators than traps at ear or ground height. Bee bowls at tassel height did not capture all species observed in corn. Therefore, to capture all of the species we observed, traps would have to be placed at all heights. Blue bowls captured more bees than white or yellow bowls; and yellow bowls captured more flies than white or blue. To provide the most efficient description of the community of insect pollinators using cornfields as a resource, we suggest sampling with BB at the height of the tassels using all three colors. Sampling using YSC is not recommended as YSC do not efficiently describe the community of insect pollinators visiting cornfields.

A criticism of using BB is that the trap is designed to be attractive to bees. It is possible that these traps are recruiting bees to the field and therefore may not represent their use of cornfields as forage or nesting sites. However, several species that we captured were observed carrying corn pollen. This suggests that the BB are not just an attractive trap, but are collecting individuals that may be using these fields as a resource.

Despite the low abundance of *A*. *mellifera* captured over the two years, this species will forage on corn [16,17]. One explanation for why we observed so few *A*. *mellifera* may be that BB are an ineffective tool for capturing this species when they are foraging. In 2013 we made visual observations of cornfields as a possible complementary sampling method. We did not observe any *A*. *mellifera* during a total of 18 hours of observation. How often *A*. *mellifera* and other pollinators forage in cornfields may be function of the surrounding landscape and the availability of more preferred forage.

We have identified a community of insect pollinators that are found in varying patterns within Iowa cornfields. To what extent these insects are using the field, as a resource for forage is still not clear. Further work is needed to elucidate why these insects are present in cornfields and what their relationship with corn pollen might be. A more detailed study that accounts for the effect of the surrounding landscape may be required to better understand the extent to which corn is used as forage for insect pollinators.

## Supporting Information

S1 File Raw Data File. Excel File containing the raw data from which our statistical analyses were performed.
(XLSX)
